# The Development and Validation of the Empathy Components Questionnaire (ECQ)

**DOI:** 10.1371/journal.pone.0169185

**Published:** 2017-01-11

**Authors:** Laurie Batchelder, Mark Brosnan, Chris Ashwin

**Affiliations:** Dept. of Psychology, University of Bath, Bath, United Kingdom; University of Bologna, ITALY

## Abstract

Key research suggests that empathy is a multidimensional construct comprising of both cognitive and affective components. More recent theories and research suggest even further factors within these components of empathy, including the ability to empathize with others versus the drive towards empathizing with others. While numerous self-report measures have been developed to examine empathy, none of them currently index all of these wider components together. The aim of the present research was to develop and validate the Empathy Components Questionnaire (ECQ) to measure cognitive and affective components, as well as ability and drive components within each. Study one utilized items measuring cognitive and affective empathy taken from various established questionnaires to create an initial version of the ECQ. Principal component analysis (PCA) was used to examine the underlying components of empathy within the ECQ in a sample of 101 typical adults. Results revealed a five-component model consisting of cognitive ability, cognitive drive, affective ability, affective drive, and a fifth factor assessing affective reactivity. This five-component structure was then validated and confirmed using confirmatory factor analysis (CFA) in an independent sample of 211 typical adults. Results also showed that females scored higher than males overall on the ECQ, and on specific components, which is consistent with previous findings of a female advantage on self-reported empathy. Findings also showed certain components predicted scores on an independent measure of social behavior, which provided good convergent validity of the ECQ. Together, these findings validate the newly developed ECQ as a multidimensional measure of empathy more in-line with current theories of empathy. The ECQ provides a useful new tool for quick and easy measurement of empathy and its components for research with both healthy and clinical populations.

## Introduction

Empathy is important for meaningful relationships in the social world. Despite its importance, there is still a lack of consensus in the field about the definition of empathy [1]. Some early researchers and theorists proposed that empathy primarily includes the cognitive ability to take another’s perspective by understanding another’s thoughts, intentions, emotions and beliefs [2,3]. Others have placed emphasis on the affective emotional response of empathy elicited by other’s feelings and emotions (e.g. [4–6]). More recently, many view empathy as a multidimensional construct comprising both cognitive and affective components [7–14]. The cognitive and affective aspects of empathy are thought to be at least partially dissociable constructs [9,15,16].

Affective empathy involves experiencing another’s feelings and emotions through recognizing, being sensitive to emotions in others and sharing the emotional experiences of others by having an appropriate affective response to the other person’s situation [9,12,14,15,17–23]. It is important that the emotional response to another’s feelings is an appropriate affective reaction to the observed emotional state [24,25]. For instance, it might not be considered affective empathy if someone reacted in a very positive way towards a friend who lost all of their money and was feeling upset. Hence the observer’s emotions and feelings must be a consequence to another’s feelings and emotions. Lawrence and colleagues [24] and Davis [26] argue that affective empathy includes: (1) parallel responses, where the observer shares the target’s emotions and feelings, and (2) reactive responses, where the observer elicits an appropriate affective reaction [16]. Taken together, these definitions suggest that the observer’s emotions and feelings must be a consequence of the target’s mental state and must also be an appropriate emotional response in order to be considered affective empathy. Sympathy is also argued to be a derivative of empathy and is defined as the awareness and sensitivity to another’s emotions and feelings, which then elicits an emotional response. However, with sympathy, the individual does not share or reciprocate the other person’s emotions and feelings and instead has an emotional response that includes feelings of sorrow, pity, comfort or emotional concern for the other person [27,28]. The recognition of another’s feelings and emotions and a sensitivity towards this are also needed for affective empathy [21,25,29–34]. Identifying and matching the facial expressions, movements, postures and vocalizations of others allows observers to resonate with other people [33,35,36].

Cognitive empathy involves the process of understanding another person’s perspective by adopting another’s point of view. The ability to take another’s perspective is consistent with what has traditionally been termed theory of mind (ToM) [3,14,37–42]. Cognitive empathy also includes the ability to judge and understand the intentions of others in order to monitor one’s own intentions [3,43]. This includes the ability to infer what others are thinking through gaze direction [3,40,41,44].

Research in individuals with psychopathy has highlighted that cognitive and affective empathy are at least partly dissociable processes. For example, research has demonstrated that individuals with psychopathy show significant deficits in affective empathy, but intact cognitive empathy [9,15,21,45–49]. Conversely, individuals with autism spectrum disorders (ASD) are argued to have intact affective empathy, but deficits in cognitive empathy [7,9,21,25,34,50]. These differing empathic profiles have been further shown in individuals who exhibit autistic traits in comparison to those who exhibit psychopathic traits in the general population [51]. Numerous neurological studies have also demonstrated distinct brain regions associated with each component of empathy [10,52]. Given the extensive evidence of a distinction between cognitive and affective components, researchers still suggest that there is still some partial overlap between the two concepts. For instance, Lamm, Batson & Decety [53] found that cognitive processes and altruistic motivational processes could modulate affective responses to others’ pain when manipulated by semantic information. Together, this evidence further implies there are two separate but interactive systems for empathy [16,54].

### Further components of empathy

Several researchers have further proposed there are further components within cognitive and affective empathy, which abilities and drives [11,55–64]. Traditionally, researchers have conceptualized empathy as an ability or skill, and lower scores on empathy questionnaires or poorer performance on behavioral tasks of empathy have been interpreted to show that an individual has low ability to empathize [65–70].

Further accounts suggest that empathy may depend on certain contexts where an individual has the drive or interest to engage with others emotionally [57,63,64,71]. More simply, there is evidence arguing that humans have a desire to interact and form meaningful social relationships [19,63,71] Rather than simply having the ability or skill to socially engage, this argument suggests that individuals are actively driven to belong and socially interact with one another [71]. In this context, ‘drive’ is defined as the strong interest, desire or behavioral tendency to emotionally engage with others and to be empathic [64,71–73]. Furthermore, drives involve motivated and goal-directed behaviors that tend to increase and operate based on positive reinforcement [73–75]. This behavior is referred to as a drive, as researchers argue that empathizing and engaging with others is a need grounded on innate mechanisms, i.e. it is not taught [63]. The basic tendency to approach or avoid environmental stimuli that underlies overall basic drives are further grounded in Pavlov’s research on reflexes through classic conditioning [76].

Recent research in individuals with psychopathy highlight this further dissociation in empathy, by suggesting significant reductions in the drive to empathize, rather than the ability, compared to controls when participants were presented videos of others experiencing pain and instructed to either try to empathize with others or to just observe [60]. Adolphs and colleagues [77] similarly examined emotion processing in a patient with rare bilateral amygdala damage, and found that the patient’s selective impairment to recognize fearful faces deteriorated when instructed to explicitly look at the eyes in the photographs, suggesting that they were driven to attend to others’ eyes. Additional evidence of further dissociations in empathy is shown through self-report. For example, Ritter and colleagues [62] assessed cognitive and affective empathy through both self-report and behavioral measures of empathy in patients diagnosed with narcissistic personality disorder (NPD) compared to patients with borderline personality disorder (BPD) and controls. Findings showed that patients with NPD exhibited an empathy profile of overestimation in affective empathy on the Interpersonal Reactivity Index (IRI; [11] but impairments on behavioral empathy tasks compared to controls. Patients with NPD also showed preserved cognitive empathy on behavioral empathy tasks but impairments on the cognitive subscales of the IRI. The authors suggest that items within the cognitive empathy subscales of the IRI tend to capture the drive to empathize aspects rather than abilities of empathy by incorporating phrases such as “I try to…” or “I tend to…” within the measure [11,62]. This may indicate that wording through self-report measures can potentially determine the ability versus drive distinction. The authors propose that individuals with NPD tend to show significant difficulties in affective empathy and report a specific motivational deficit in cognitive empathy. It is worth noting that the authors further suggest that items for the affective empathy subscale of the IRI tend to assess abilities in affective empathy rather than drives in comparison to the cognitive empathy subscales. To date, current measures of empathy do not fully index all of these further components of empathy, revealing a gap between the current ideas about empathy and the commonly used self-report measures that index empathy.

### Measuring empathy through self-report

Numerous self-report measures of empathy have been developed and validated over the years. However, many measures have been critiqued because the ambiguous definition of ‘empathy’ has led to inconsistent interpretations of findings within the literature [16,28]. Some empathy measures also tend to use different definitions that do not relate to current theoretical ideas about empathy [16,78,79]. To date, there are no assessments that measure all four components of empathy. For instance, some self-report measures only rely on one aspect of empathy rather than taking into consideration the multidimensional nature of empathy. One of the very first and most widely used self-report measure of cognitive empathy is the Hogan’s Empathy (EM) Scale [2]. Although the scale is argued to measure cognitive empathy [2,80], further psychometric analysis of the HES showed poor psychometric analysis [81]. Others have argued that the HES may measure general social skills, rather than cognitive empathy [7,24].

Other measures focus on measuring the affective component of empathy. The Questionnaire Measure of Emotional Empathy (QMEE) specifically aim to measure an individual’s tendency or drive to respond to another’s emotions and feelings [82]. Mehrabian and Epstein [82] aim capture empathy exclusively as an emotional experience by defining it as, “a vicarious response to the perceived emotional experiences of others” ([82] pg. 525). However, others speculate that this measure may be assessing people’s emotional reactions to their general environment, rather than in response to other’s emotions [11,83].

The Interpersonal Reactivity Index (IRI) [11,83] is one of the most commonly used self-report questionnaire to-date and was developed to assess the cognitive and affective empathy simultaneously. It measures both cognitive and affective empathy across four different subscales: perspective-taking; fantasy; empathic concern; and personal distress. The perspective taking and fantasy subscales index cognitive empathy, and empathic concern and personal distress measure affective empathy [11,83]. The IRI has been shown to be well-validated [11,84–87] and has strong test-retest reliability [11,88]. However, others argue that some of the subscales do not directly reflect empathy. For example, the fantasy subscale may relate more to imagination and emotional self-control than empathy [7,24,89]. Further research shows that the four-factor model previously validated by Davis [11,83] is a poor fit for the IRI (e.g. [90,91]). Cliffordson [92] suggested that the personal distress subscale might not be a key component of empathy after conducting a factor analysis of the IRI and found that the four-factor solution was not resolved. These studies question whether the underlying components of the IRI accurately reflect the key frameworks about empathy, including even the cognitive and affective components [7,24] Evidence of further components of empathy captured through specific wording in the IRI have been suggested by Marcoux et al. [59] and Ritter et al. [62]. However there is a need to explicitly assess and validate further components of cognitive and affective empathy, and further dissociation between ability and drive within these components.

Another popular self-report questionnaire of empathy is the Empathy Quotient (EQ), which was developed to index empathy in both the general population and in different clinical samples, such as autism spectrum disorders (ASD) [7]. The EQ was validated on 197 controls and 90 individuals with ASD and showed reliability between both controls and clinical groups [7]. The EQ measures empathy through a total score without delineating cognitive and affective components [7,16] The lack of scores provided for further components limits how informative the results can be and how well it aligns with theoretical frameworks about empathy. Although further assessment of the EQ’s factor structure has revealed that the EQ includes cognitive, affective and broader social skills factors in its items [24,61], once again suggesting that empathy is a multidimensional construct involving cognitive and affective aspects. However, it is unclear whether there are further aspects of ability and drive measured through the EQ. For example, when examining the validity of the EQ, Muncer and Ling [61] speculated that the affective component of empathy is thought to relate more to the drive to empathize, rather than the ability to empathize, when identifying emotions and mental states of others [11,93]. Based on current empathy scales and evidence, there is a need for an explicit measure that captures current theoretical ideas about empathy.

### Sex differences in empathy

Evidence has shown there are sex differences in empathy, with females showing superiority compared to males. Multiple studies highlight female superiority on self-reported empathy measures, such as the EQ [7,94]. Interestingly some research suggests that females tend to self-report higher on affective empathy in comparison to males, but exhibit minimal or lack of sex differences on the cognitive component of empathy (e.g. [11,61,95]). For instance, Muncer and Ling [61] reported a particularly strong sex difference for an ‘emotional reactivity’ component of the EQ. Emotional reactivity is part of affective empathy and is thought to relate more to the drive to empathize, rather than the ability to empathize, when identifying emotions and mental states of others [11,61,93]. The authors proposed this finding might reflect a greater drive to empathize in females compared to males. This indicates that females report having a greater interest or drive to recognize and be sensitive to others’ emotions, which then allows them to affectively react to other’s feelings and emotions. Similar findings from dispositional measures of empathy indicate that females tend to report higher empathy compared to their male counterparts [96]. One interpretation of these findings is that there may be an over-estimation of one’s empathic behaviors [97–101]. This dissociation could in part be due to a social desirability response bias [78]. Furthermore, females may exhibit a greater drive to empathize based on their reported beliefs about their own empathy [96,102]. For instance, Eisenberg and Lennon [103] suggest that females may be motivated by gender role expectations, as cultural stereotypes hold that females tend to be more empathic and overall more social compared to males [55,102–104]. Based on current findings within the literature, this suggests that a fuller understanding of sex differences in empathy will be obtained through an assessment of both cognitive and affective empathy in addition to considering drive and ability, which requires the development of a new scale.

The main aim of the current study was to develop and validate the Empathy Components Questionnaire (ECQ), a new self-report empathy measure that aimed to include further components more in-line with current theories about empathy. These components included cognitive and affective aspects, along with the ability and the drive to empathize within each of these components. This research consisted of two studies. Study one aimed to develop a quick and easy to administer instrument that measures empathy and its proposed further components. Study one used a principal component analysis (PCA) to identify the factor structure of the ECQ and reduce its length to its core components. Study two included a second sample of participants that completed the refined ECQ. A confirmatory factor analysis (CFA) was used to verify the factor structure generated by the PCA in Study one. Study two also assessed sex differences across all components of empathy. An independent measure of social behavior was also included in study two to examine convergent validity of the ECQ.

It was hypothesized that there would be components extracted from the ECQ capturing cognitive and affective components of empathy, along with potential further ability and drive dissociations within each component. This would produce a model consisting of cognitive ability, cognitive drive, affective ability, and affective drive, in-line with recent theories of empathy [11,19,55,57–64]. It was also predicted that the ECQ would show good reliability, since the items included in the initial ECQ were taken from previously developed and validated empathy questionnaires. It was expected that this multidimensional model of empathy would be confirmed through CFA within an independent sample. As females tend to report higher self-report empathy in comparison to males as shown within the literature (e.g. [7,105]), sex differences on the ECQ components were also examined. Based on previous research, it was predicted females would report higher affective empathy compared to males. However, given that there tends to be smaller sex differences reported on measures of cognitive empathy [6,11,106], it was expected there would be minimal sex differences on the cognitive components of the ECQ. It was also hypothesized that some components would predict scores on an independent measure of social behavior, showing good convergent validity of the ECQ.

## Study One

### Methods

#### Participants

The sample included 101 University students and staff (mean age = 20.31; SD = 1.90) consisting of 66 females (mean age = 20.26, SD = 1.92) and 35 males (mean age = 20.40, SD = 1.90). They were recruited by opportunity sample from the University of Bath through various online social networks (e.g. Facebook, Twitter, etc), active personal recruitment on campus, electronic noticeboards on the University websites, and using campus noticeboards from a variety of departments to provide a wide academic background (42.6% Humanities, 40.6% Sciences, 7.9% Other, 8.9% Not reported). The majority of the sample (74.2%) identified as White British, and the remaining participants either reported as Asian or Asian British (3.0%), of a mixed ethnicity (2.0%), of any other ethnicity (2.0%), or preferred not to say (18.8%). 89 participants also reported English as their native language. None of the participants reported having a clinical diagnosis, which was an exclusion criterion.

#### Materials

The ECQ was constructed to measure further aspects of empathy including cognitive and affective components, and to further delineate the drive and ability within these components. The first step was to choose a number of items that indexed cognitive or affective components of empathy and determine how effectively they indexed cognitive and affective empathy. Potential items for the ECQ were derived from five popular questionnaires of empathy within the field which incorporate items measuring both cognitive and affective empathy, in order to benefit from the strength of well-validated and established measures [16,107]. These included 22 items selected and rated from the EQ-short [108], which includes items derived from the original and longer EQ [7], 28 items selected and rated from the IRI [11]; 8 items selected and rated from the Empathy Subscale of the Emotional Quotient Inventory (EQ-i) [109,110], and 31 items selected and rated from the Questionnaire of Cognitive and Affective Empathy (QCAE) [16], which includes items derived from the EQ (15 items) [7], the Hogan Empathy Scale (HES) (two items) [2], the Empathy Subscale of the Impulsiveness-Venturesomeness-Empathy Inventory (IVE) (8 items) [111] and the IRI (6 items) [11]. The 22 item short form of the EQ was included rather than the original larger EQ, because the EQ-short incorporates essential empathy items without any non-essential filler items [108]. This resulted in a total of 89 items proposed to index empathy.

The validity of these 89 empathy items was initially evaluated by four researchers from the Department of Psychology who judged whether each item measured either cognitive empathy (perspective-taking), affective empathy (recognizing, being sensitive to, sharing and responding with appropriate emotions), or neither of these (e.g. sympathy, fantasy, personal distress, or broader social skills). The raters were provided with definitions of each category based on those provided by Lawrence et al. [24] and rated each of the 89 items independently. If all four raters agreed on the choice of cognitive or affective empathy for a given item, the item was included as part of the ECQ for further evaluation. In cases of disagreement, the item was omitted. This resulted in 39 items identified as accurately measuring either cognitive (21 items) or affective (18 items) empathy.

Items were further categorized into ability and drive within each component. The same four researchers predicted that items measured one of the four components: cognitive ability, cognitive drive, affective ability, or affective drive. These items were categorized based on the following definitions. Cognitive ability was defined as the skill, capacity or potential in perspective-taking and to adopt another’s point of view; cognitive drive was defined as the motivated interest or tendency in perspective-taking and to adopt another’s point of view; affective ability was defined as the skill, capacity or potential in recognizing, being sensitive to and sharing others’ emotional experiences; and affective drive was defined as the motivated interest or tendency in recognizing, being sensitive to and sharing others’ emotional experiences. These definitions were developed based on previous literature suggesting that individuals may vary in their abilities to empathize compared to their drive to empathize, such as individuals with ASD [57,60,64,112]. Similar to the initial item selection process of the 89 items, the four researchers rated each of the 39 items independently based on definitions for each component. If all four raters agreed on the choice for a given item, it was included as part of the ECQ for further evaluation. In rare situations of disagreement, researchers re-focused on the further definitions of empathy to allow for further assessment and discussion. Raters agreed to still include these items in this instance, as it was appropriate to be open in investigating additional components of empathy, particularly since these items were allocated to either cognitive or affective empathy during the initial selection process. It was also important to be open to potential other components within either the cognitive or affective component that might arise through the PCA. After rating these items, 39 items were predicted to capture cognitive and affective empathy, and more specifically: cognitive ability (10 items), cognitive drive (11 items), affective ability (7 items), or affective drive (11 items).

A four-point response scale (1 = Strongly Disagree to 4 = Strongly Agree) was chosen in order to capture the differences in responses. The current study incorporated a four-point Likert-type scale to avoid true neutral responses and to avoid social desirability bias [113]. This was similar to the EQ [7] and the QCAE [16]. There were five negatively worded items, which were reversed scored (1 = Strongly Agree to 4 = Strongly Disagree). The order of the items was then randomized to produce the first version of the ECQ.

#### Procedure

Ethical approval for the present study was obtained from the Psychology Department Research Ethics Committee of the University of Bath, and all participants gave informed consent.

All participants completed Study one online via Bristol Online Survey (BOS). Participants viewed each question individually on a computer screen, and then rated the scale about how much they agreed or disagreed with each statement. There was no time limit, and participants took approximately 10 minutes to complete the study. After testing was completed, all participants were debriefed on the nature and purpose of the study.

#### Data analysis strategy

In order to explore the factor structure of the initial version of the ECQ, a PCA using orthogonal varimax rotation was run using SPSS statistical software (SPSS Inc., Chicago, IL). PCA is a statistical variable reduction technique used to explore and analyze various dimensions within a dataset and extract meaningful underlying variables [114–117]. Traditional factor analysis (exploratory factor analysis) tends to differ in its technique compared to PCA in that factor analysis estimates factors and focuses on various assumptions in these predictions by determining the number of latent variables that account for shared variance [117,118]. From a statistical point of view, PCA is a technique used to measure the structure of ECQ variables and to reduce these variables into components. Factors are groups of correlation coefficients between subsets of variables potentially measuring similar constructs within a correlation matrix [114,115]. It is then important to assess how these factors cluster together in a significant way, along with explaining the maximum amount of variance. These processes are argued to be similar in nature apart from the preparation of the observed correlation matrix and underlying theory [117]. For instance, components derived from PCA are aggregates of correlated variables to explain underlying processes, whereas factors in the EFA are causal [117]. However, some evidence suggests that both procedures exhibit similar factor patterns [114–116,119–121]. Thus both techniques specifically examine underlying dimensions of a dataset, and arguably PCA is more useful in reducing multiple observed variables into fewer key components that capture the overall variance [114,115,117,118]. As a result, PCA was used for the interpretation of the structure model and dimensionality of the ECQ in the current study because it was necessary to examine and explore whether items within the current measure can be reduced to index the key theoretical components of empathy to account for its maximum variance [24,61]. While it was predicted the ECQ would index cognitive and affective components of empathy, a PCA was specifically implemented to also examine other potential components. Hence, this study was open to exploring other potential components that can be reduced and estimated in order to account for the overall variance within the ECQ [117,118,122]. This was done to determine the exact factor structure of the ECQ and to see whether these 39 items can be reduced to core questions assessing essential aspects of empathy. A PCA can also identify associated underlying concepts accounting for most of the variance and omits redundant or unnecessary items accounting for less variance within a questionnaire [114,115,117,123,124]. Analyses were also conducted to compare scores on components of the ECQ across males and females.

### Results

There was no difference in age between males (mean = 20.40, SD = 1.90) and females (mean = 20.26, SD = 1.92), *t* (99) = 0.36, *p* = 0.72.

#### Principal Component Analysis (PCA)

There were no missing values in the data. None of the ECQ items had skew of a magnitude of +/-2.0 or higher, which is the recommended cut-off criteria [125,126]. A value of +/-3.0 or more is the excess kurtosis cut-off and indicates a large deviation from normality [125]. These were also the only items to have kurtosis values of more than 2.0: actual values 2.70 and 2.62. Mahalabonis distance values were calculated for each participant for the initial ECQ in order to check multivariate normality in preparation for the PCA. All items of the ECQ were entered as predictor variables. Employing a χ^2^ of 72.06 (*df* = 39) and a significance criterion *p*-value of 0.001 resulted in the identification of no significant multivariate outliers. Hence all 101 cases were included for PCA.

Inspection of the correlation matrix revealed items from the initial ECQ correlated fairly well, with minimum correlation coefficients of 0.10. Multi-collinearity was also not found based on cut-off criteria of 0.90 [114,115]. The Kaiser-Meyer-Olkin measure of sampling adequacy was 0.71, above the recommended value of 0.60 [127–129], and the Bartlett test of sphericity was highly significant (1852.64, *p* < 0.001), indicating that PCA is appropriate for this dataset [114–116,118,130]. Communalities between items were also above 0.40, further confirming that each question shared some common variance [114–116,118].

The PCA using orthogonal varimax rotation revealed the presence of eleven components, with eigenvalues exceeding a Kaiser’s criterion of 1, explaining 69.20% of the total variance. Orthogonal rotation was chosen based on the theoretical assumption cognitive and affective empathy are partially dissociable components. Varimax rotation also allows for easy interpretation of factor loadings as it maximizes the amount of variance of items and leads to a smaller number of large loadings for each factor [114,115,127,131,132]. To better interpret these factor loadings from the orthogonal varimax rotation, a scree plot was also used for the current analysis [114,115,133]. The scree plot showed that there were three inflexion points: one at eigenvalue six, the second at eigenvalue eight and the third at eigenvalue eleven. Variances of each component were then examined, and it was revealed that only six components accounted for over 5% of the proportion of variance [118]. After careful examination of each component and their underlying items, these six items were retained for rotation and further interpretation, explaining 47.50% of the total variance.

Double loadings were also allocated on the basis of content and their strength in component loading. Only items loadings above 0.40 were included in the scale. Component loadings ranged from 0.40 to 0.86. It was important to examine if there was any theoretical overlap between any of the items. Components five and six both contained items interpreted by the authors as measuring cognitive empathy, whether overall or in specific contexts. These two factors were also highly positively correlated (*r* = 0.52, *p <* 0.00001), and only a small number of items loaded onto the sixth factor (3 items). Due to this significant overlap between the components and the small number of items, it was decided to combine them into one overarching component. This left five components with at least three items and loadings equal to, or above, 0.40. These five components were defined as follows based on previous theories and literature suggesting further components of empathy (e.g. [11,19,55,57–64]): affective reactivity, cognitive drive, affective ability, affective drive, and cognitive ability (see Table 1). It is worth noting that initially affective reactivity was proposed to be a facet of affective drive, however results and further theory suggest that affective drive and affective reactivity are separate components.

**Table 1:** Final rotated component factor loadings from the initial ECQ using PCA in 101 participants.

Item	Question	Affective Reactivity1	Cognitive Drive2	Affective Ability3	Affective Drive4	Cognitive Ability5	Social Perspective-taking6
**1**	**ECQ18**. It affects me very much when one of my friends is upset. (IVE)(+)	**0.82**					
**1**	**ECQ20**. I get very upset when I see someone cry. (IVE)(+)	**0.76**					
**1**	**ECQ14**. I am happy when I am with a cheerful group and sad when others are glum. (IVE)(+)	**0.73**					
**1**	**ECQ24**. The people I am with have a strong influence on my mood. (IVE)(+)	**0.70**					
**1**	**ECQ2**. It worries me when others are worrying and panicky. (IVE)(+)	**0.62**					
**1**	**ECQ21**. I tend to get emotionally involved with a friend’s problems. (EQ)(+)	**0.61**					
**1**	**ECQ31**. Sometimes I don’t feel sorry for other people when they are having problems (IRI)(-)	**0.61**					
**1**	**ECQ12**. I care what happens to other people. (EQ-i empathy subscale) (+)	**0.47**					
**2**	**ECQ29**. I try to look at everybody’s side of a disagreement before I make a decision. (IRI)(+)		**0.75**				
**2**	**ECQ30**. I sometimes try to understand my friends better by imagining how things look from their perspective. (IRI)(+)		**0.73**				
**2**	**ECQ9**. Before criticizing someone, I try to imagine how I would feel if I were in their place. (IRI)(+)		**0.67**				
**2**	**ECQ23**. I can usually appreciate the other person’s viewpoint, even if I do not agree with it. (EQ)(+)		**0.64**				
**2**	**ECQ19**. When I’m upset with someone, I usually try to ‘put myself in his shoes’ for a while. (IRI)(+)		**0.57**				
**3**	**ECQ27**. Other people tell me I am good at understanding how they are feeling and what they are thinking. (EQ)(+)			**0.86**			
**3**	**ECQ11**. Friends usually talk to me about their problems as they say that I am very understanding. (EQ)(+)			**0.85**			
**3**	**ECQ3**. I can tune into how someone feels rapidly and intuitively. (EQ)(+)			**0.62**			
**3**	**ECQ28**. I’m sensitive to the feelings of others. (EQ-i empathy subscale)(+)			**0.45**			
**4**	**ECQ26**. I avoid hurting other people’s feelings. (EQ-i empathy subscale)(-)				**0.76**		
**4**	**ECQ5**. I always try to consider the other fellows’ feelings before I do something. (HES)(+)				**0.68**		
**4**	**ECQ34**. Before I do something, I try to consider how my friends will react. (HES)(+)				**0.60**		
**4**	**ECQ4**. I really enjoy caring for other people. (EQ)(+)			**0.40**	**0.53**		
**5**	**ECQ1**. I am good at predicting what someone will do. (EQ)(+)					**0.70**	
**5**	**ECQ33**. I can easily work out what another person might want to talk about. (EQ)(+)					**0.70**	
**5**	**ECQ37**. I can pick up quickly if someone says one thing but means another. (EQ)(+)					**0.48**	
**5**	**ECQ25**. I find it easy to put myself in somebody else’s shoes. (EQ)(+)		**0.44**			**0.46**	
**6**	**ECQ6**. I can sense if I am intruding, even if the other person does not tell me. (EQ)(+)						**0.75**
**6**	**ECQ36**. I am quick to spot when someone in a group is feeling awkward or uncomfortable. (EQ)(+)						**0.71**
**6**	**ECQ16**. I can tell if someone is masking their true emotion. (EQ)(+)						**0.60**

#### Sex differences

**Fig 1:**
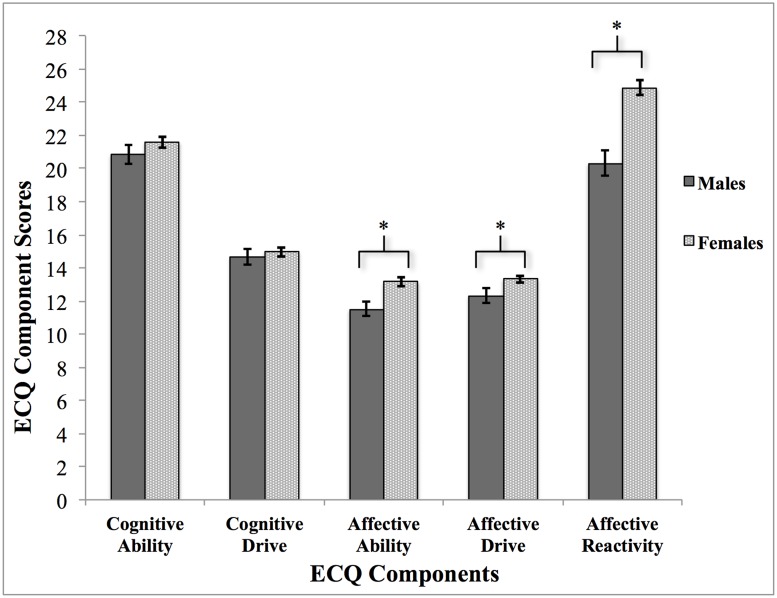
Assessment of sex differences across the preliminary five components extracted from the ECQ in 101 participants.

Sex differences in the ECQ were examined on total ECQ scores through an independent t-test. There was a significant difference in total empathy scores between males (mean = 79.69, SD = 9.65) and females (mean = 87.94, SD = 8.58), *t* (99) = -4.41, *p* < 0.0001. In order to assess sex differences across all five components in further detail, a between-subjects MANOVA with sex (male versus female) as the independent variable and the preliminary five ECQ components as the dependent variables was also conducted (Table 2). Results of the MANOVA revealed a significant interaction between sex and the five ECQ components at a multivariate level, Hotelling’s *T* (0.36), *F* (1, 99) = 6.82, *p* < 0.0001, partial eta squared = 0.26. Post-hoc univariate analyses revealed a statistically significant effect between sex and scores on affective ability (*F* (1, 99) = 12.03, *p ≤* 0.001, partial eta squared = 0.11), affective drive (*F* (1, 99) = 5.42, *p* = 0.02, partial eta squared = 0.05), and affective reactivity (*F* (1, 99) = 30.54, *p <* 0.0001, partial eta squared = 0.24). There were no statistically significant differences between females and males on cognitive ability (*F* (1, 99) = 1.28, *p* = 0.26) or cognitive drive (*F* (1, 99) = 0.29, *p* = 0.59) (see Fig 1).

**Table 2:** Total ECQ and component mean scores for 101 males and females.

Measure	Males Mean (SD)	Females Mean (SD)
**Total ECQ**	79.69 (9.65)	87.94 (8.58)
**Cognitive**	35.54 (5.18)	36.56 (4.21)
Cognitive Ability	20.86 (3.53)	21.59 (2.86)
Cognitive Drive	14.69 (2.90)	14.97 (2.29)
**Affective**	44.14 (6.84)	51.38 (5.90)
Affective Ability	11.51 (2.45)	13.17 (2.18)
Affective Drive	12.31 (2.59)	13.33 (1.78)
Affective Reactivity	20.31 (4.41)	24.88 (3.69)

### Discussion

The results from Study one revealed the ECQ had a model consisting of the five factors of cognitive ability, cognitive drive, affective ability, affective drive, and a fifth factor named affective reactivity. These findings are consistent with recent theory and research in the field, which conceptualizes empathy as comprising of multiple components [11,19,55,57–64]. Affective reactivity was extracted as a fifth factor and is consistent with the proposal that appropriately responding and reacting to another’s emotional experiences is a key part of empathy [7,8,12–14,19,23,24,61]. This separate factor of affective reactivity is independent from affective drive and affective ability, which is consistent to the factor assessment and interpretation of items within the EQ [61]. Self-report affective reactivity tended to be best reflected with questionnaire items loading onto this factor with situations such as one becoming happy with a cheerful group of people, upset when someone is crying, or genuinely caring for others. This is in-line with evidence suggesting affective empathy comprises of an emotional response in which an individual reacts to another’s feelings or emotion by sharing the emotional experience of another through synchronizing or complementing these emotions or feelings [12–14,24,29,56,80,134]. Interestingly, this factor also included an item from the IRI empathic concern subscale, with the item stating, ‘Sometimes I don’t feel sorry for other people when they are having problems’. Davis [11,83] included the empathic concern subscale in the IRI to assess tendencies to experience feelings of compassion and concern for others whom are undergoing negative experiences, such as pain or sadness. This could suggest that the experience of concern and caring for others may relate to the ways in which individuals appropriately experience and respond to others’ emotions in various contexts, such as sympathy. This could suggest that the tendency for an individual to experience concern for others overlaps with one reacting to another’s perceived emotional state. It is worth noting that it was initially predicted items from the initial ECQ would be reduced to four components examining abilities and drives within cognitive and affective empathy, however analysis was also open for further potential components of empathy. This component was interpreted as explicitly measuring the emotional response itself and not necessarily the ability or the drive to so. It could be that the tendency to synchronize or complement another’s feelings or emotions may be the outcome of having the ability or drive in recognizing or be sensitive towards other’s emotions, such as through facial expressions, postures or movements or voices, another aspect of affective empathy [16,32,53,56,83,135–137]. However, there is still a need for the affective ability and affective drive components to be considered as well, as these components are shown to be needed for the empathic process [9,11,16,32,83].

Sex differences were also examined across the preliminary components and revealed females scored significantly higher than males on the affective empathy components. This suggests that females self-reported higher levels of abilities and drives in recognizing and being sensitive to other’s feelings and emotions, as well as emotionally reacting to others’ emotions. These results are consistent with previous research showing females score significantly higher than males on self-report measures of empathy [7,16,24,96,105,106]. On the other hand, there were no significant sex differences on both cognitive empathy components of the initial ECQ. This lack of sex difference in cognitive empathy has been documented within some of the literature [6,11,106], although sex differences tend to exist in empathy due to females reporting greater emotional reactivity to other’s feelings and experiences.

## Study Two

### Introduction

While the results from Study one showed a five-factor model of the ECQ, some issues remained about the nature of the wording in the items. Some items were too similar in wording, and there was an imbalance of positive and negative worded questions. The majority of questions within each factor consisted of positively worded questions. A more balanced mixture of positively and negatively worded questions would avoid the risk of response bias and social desirability [138]. To avoid repetitive wording, it is important to better distinguish items on their respected factors from the remaining factors by refining these items using words that better reflect the content each factor represents [11,139,140]. In addition, all of the participants in Study one were recruited from the University of Bath, which could limit generalizations of the current findings. However the sociodemographic profile of the sample in Study one is consistent with other reported sociodemographic profiles in current empathy questionnaire literature [16,107]. In order to overcome this limitation, there was a need to test the ECQ in a larger, more generalized sample comprised of participants with a variety of professional and academic backgrounds in and outside the University of Bath. This would increase the external validity of the research.

To address these concerns, the aims of Study two were to refine the wording in the items of the ECQ and to run a CFA to test the model fit in a larger independent sample. Study two also aimed to further assess differences on the five components and to establish convergent validity of the ECQ with an independent measure of social behavior. For this purpose, the Social Interest Index- Short Form (SII-SF) was included to specifically measure one’s overall drive and willingness to be social. It was expected the ECQ with refined wording would show the same five-factor model fit, which was found in Study One. It was also hypothesized that females would score higher on the affective components compared to their male counterparts, but score similarly on the cognitive components, which was found in Study one. It was hypothesized drives in cognitive and affective empathy, as well affective reactivity, would positively correlate with scores on the SII-SF [141].

### Methods

#### Participants

The sample consisted of 265 adults recruited via opportunity sampling within the University of Bath campus and the wider general adult community in the UK. Recruitment was undertaken through campus noticeboards, online research recruitment sites (e.g. Psychology Research on the Net, In Mind and Social Psychology Network) and through online social networks (e.g. Facebook, Twitter and Reddit). Forty-five of the participants were removed because they self-reported having a psychiatric diagnosis, which was an exclusion criteria. Two participants were also removed on the basis of incomplete data sets. A further seven outliers (two unidimensional outliers and five multidimensional outliers) were removed based on calculated distances outside of the normally distributed data. This left a total of 211 participants whose data was included in the final dataset (mean age = 27.75, SD = 8.75). This sample was comprised of 116 females (mean age = 29.21, SD = 9.95) and 95 males (mean age = 25.98, SD = 6.64), with approximately half being University students (N = 100) and the others being non-students in employment (N = 111). Students were also recruited from a variety of departments and faculties to provide a wide academic background (60.0% Humanities, 37.0% Sciences, 3.0% Other). 195 participants also reported English as their native language. Demographics of participants for Study two are outlined in Table 3.

**Table 3:** Group demographics in Study Two.

Demographics	Females (N = 116)	Males (N = 95)	*t*	*p-value*
Age	29.21 (9.95)	25.98 (6.64)	-2.71	0.01
Students	56 (48.3%)	46 (48.4%)		
Non-Students	60 (51.7%)	49 (51.6%)		

#### ECQ item refinement

The wording of the ECQ items was refined for three main reasons. Firstly this would align questions better with the components found in Study one and to further distinguish between them. Clearer questions intended to better reflect specific components, particularly highlighting dissociations between ability and drive, would allow for less ambiguity [142]. The refined ECQ also included both positively and negatively worded questions to help reduce response bias [143–146]. Thirdly, it was predicted rewording the questions would help reduce repetitive wording within the questions, since similar phrases were used across different questions taken from various empathy measures. All three of these revisions were thought to strengthen the ECQ in assessing the various components of empathy identified by the PCA in Study One [11,138–140,145].

The refined ECQ consisted of a total 30 items. The original version of the ECQ contained 28 items taken from the initial ECQ questionnaire used as core items [11,83] to be refined and adapted. Two new items were generated and included here that were proposed to be related to one of the five empathy components in order to balance the number of items within each component. This was to provide similar representation of each of the five components [147]. The first item, ‘I am good at responding to other people’s feelings’ was created to assess the ability to recognize and be sensitive to others’ emotions. The affective ability component comprised of only four items with three negatively worded items after the refinement process, hence it was important to include an item that fully captures the nature of this component and to better balance the number of positive and negative worded questions. In developing this item, wording was based on the definition of affective ability, which was defined as the skill, capacity or potential in recognizing and sharing others’ emotional experiences. The second item, “When talking with others, I am not very interested in what they might be thinking” was created to examine the drive to take another’s perspective. This additional item was included to further capture cognitive drive. The cognitive drive component also only included four items that were all positively worded, so it was important to also include a negatively worded item to better balance the number of positive and negative worded questions. The items were initially developed by one researcher and then sent to two other researchers to be reviewed and refined.

ECQ items were refined based on definitions of abilities and drives, in order to align items even better to components of empathy. Both positively and negatively words were also used in the rewording of the items. Positive words measuring the construct of ability in the present rewording the items included: well, able, good and success. Negative words measuring the construct of ability in the rewording of items in the present research included: poor, not very good, unsuccessful and unable. Positive words measuring the construct of drive in the present rewording of the items included: desire, interested, motivate, tend, strive, like, enjoy and willing. Negative words measuring the construct of drive in the rewording of the items included: uninterested, avoid, unaffected and not motivate. During the refinement process, three researchers compared the original questionnaire items with the proposed refined items including the key ability and drive words. The questions were then reworded to include these key item words. After the rewording of the items, the 30 items were rated on how well the items from the refined ECQ with the new words for ability and drive matched the original definition provided for each component by three researchers. In cases of disagreement between the raters, items were further re-constructed, rated and discussed (see Table 4).

**Table 4:** The ECQ in the current study including both refined items compared to their original wording and two new items.

Original Questions	Refined Questions
**Factor One: Affective Reactivity**	
It affects me very much when one of my friends is upset (IVE)	When someone seems upset, I am usually uninterested and unaffected by their emotions. (-)
I get very upset when I see someone cry (IVE)	When someone is crying, I tend to become very upset myself. (+)
I am happy when I am with a cheerful group and sad when others are glum (IVE)	I am not always interested in sharing others’ happiness. (-)
The people I am with have a strong influence on my mood (IVE)	Others’ emotions do not motivate my mood. (-)
It worries me when others are worrying and panicky (IVE)	I tend to panic when I see others who are panicked. (+)
I tend to get emotionally involved with a friend’s problems (EQ)	I avoid getting emotionally involved with a friend’s problems. (-)
Sometimes I don’t feel sorry for other people when they are having problems (IRI)	I feel pity for people I see being bullied. (+)
I care what happens to other people. (EQ-i empathy subscale)	I like to know what happens to others. (+)
**Factor Two: Cognitive Drive**	
I try to look at everybody’s side of a disagreement before I make a decision (IRI)	I enjoy debates because I like to take different perspectives. (+)
I sometimes try to understand my friends better by imagining how things look from their perspective (IRI)	I like trying to understand what might be going through my friends’ minds. (+)
Before criticizing someone, I try to imagine how I would feel if I were in their place (IRI)	I strive to see how it would feel to be in someone else’s situation before criticizing them. (+)
I can usually appreciate the other person’s viewpoint, even if I do not agreewith it (EQ)	I take an interest in looking at both sides to every argument. (+)
When I’m upset with someone, I usually try to ‘put myself in his shoes’ for a while (IRI)	I am uninterested in putting myself in another’s shoes if I am upset with them. (-)
NEW ITEM: When talking with others, I am not very interested in what they might be thinking. (-)	
**Factor Three: Affective Ability**	
Other people tell me I am good at understanding how they are feeling and what they are thinking (EQ)	I am not very good at helping others deal with their feelings. (-)
Friends usually talk to me about their problems as they say that I am very understanding (EQ)	My friends often tell me intimate things about themselves as I am very helpful. (+)
I can tune into how someone feels rapidly and intuitively (EQ)	I don’t intuitively tune into how others feel. (-)
I’m sensitive to the feelings of others (EQ-i empathy subscale)	I am poor at sharing emotions with others. (-)
NEW ITEM: I am good at responding to other people's feelings. (+)	
**Factor Four: Affective Drive**	
I avoid hurting other people’s feelings (EQ-i empathy subscale)	I am not interested in protecting others, even if I know they are being lied to. (-)
I always try to consider the other fellows’ feelings before I do something (HES)	When I do things, I like to take others’ feelings into account. (+)
Before I do something, I try to consider how my friends will react (HES)	I avoid thinking how my friends will respond before I do something (-)
I really enjoy caring for other people (EQ)	I have a desire to help other people (+)
**Factor Five: Cognitive Ability**	
I am good at predicting what someone will do (EQ)	I’m not very good at predicting what other people will do. **(-)**
I can easily work out what another person might want to talk about (EQ)	During a conversation, I’m not very good at figuring out what others might want to talk about (-)
I can pick up quickly if someone says one thing but means another (EQ)	I am usually successful in judging if someone says one thing but means another **(+)**
I find it easy to ‘put myself in somebody else’s shoes’(EQ)	I am not very good at ‘putting myself in others’ shoes’ **(-)**
I can sense if I am intruding, even if the other person does not tell me (EQ)	I am good at sensing whether or not I am interrupting a conversation **(+)**
I am quick to spot when someone in a group is feeling awkward or uncomfortable(EQ)	I do well at noticing when one of my friends is uncomfortable **(+)**
I can tell if someone is masking their true emotion (EQ)	I am not very good at noticing if someone is hiding their emotions **(-)**

+ Refer to positively worded questions,

- Refer to negatively worded questions

Note: Abbreviations in brackets refer to original empathy measures the items were originally taken in developing the ECQ; EQ = Empathy Quotient; IRI = Interpersonal Reactivity Index; IVE = Impulsiveness-Venturesome-Empathy Inventory; EQ-i = Emotional Quotient Inventory; HES = Hogan Empathy Scale

A four-point response scale (1 = Strongly Disagree to 4 = Strongly Agree) was used to measure the differences in responses. Negatively worded items were reversed scored (1 = Strongly Agree to 4 = Strongly Disagree). The DVs were scores for each component of the ECQ and the total cumulative ECQ score.

#### Measures

In addition to the refined ECQ, participants in Study two completed the following measure for the purposes of convergent validity.

#### Social Interests Index- Short Form (SII-SF) [141]

The short form of the Social Interests Index (SII-SF) is a 14-item self-report measure that assesses a sense of social feeling toward friendship, love and work, with questions including ‘My friends are very important to me’ and ‘I have warm relationships with some people’ [141]. This measure was included as it is not an explicit measure of empathy but of being socially oriented, which would be expected to relate to empathy. The SII-SF employs a five-point Likert scale ranging from 1 ‘not at all like me’ to 5 ‘very much like me.’ The SII-SF total score ranges from 14 (low social interest) to 70 (high social interest). The DV was the total cumulative social interest score.

#### Procedure

Ethical approval for the present study was obtained from the Psychology Department Research Ethics Committee of the University of Bath, and all participants gave informed consent.

Participants completed Study two online via Bristol Online Survey (BOS). Similarly to study one, participants viewed each question individually on a computer screen, and then rated the scale about how much they agreed or disagreed with each statement. There was no time limit for each question, and participants took approximately 15 minutes to complete the study. After testing was completed, all participants were debriefed on the nature and purpose of the study.

#### Data analysis strategy

CFA was used to confirm the five-factor structure of the ECQ in this independent sample. CFA theoretically differs from exploratory factor analysis or PCA and is more useful in testing a specific theory because the theory is directly tested by the analysis [117,118,122]. CFA is a method used to test a specific hypothesis a priori by assessing whether there is a relationship between observed variables and their underlying latent constructs [148]. This method is particularly useful for questionnaire development in refining items and assessing a questionnaire’s construct validity. In addition, scores on the five components of the ECQ were compared between males and females. A two-step hierarchical multiple regression was also implemented to assess convergent validity of the ECQ.

### Results

#### Confirmatory Factor Analysis (CFA)

There were no missing data, and univariate and multivariate outliers that were three standard deviations or greater away from the mean were excluded (3.32% of the total dataset). The five-factor structure suggested by the PCA in Study one was tested using a CFA by utilizing Analysis of Moment Structures (AMOS 7.0) [117,149,150]. The present sample size of 211 participants was considered suitable for undertaking a CFA based on the recommendations of. Tabachnick & Fidell [117], Myers, Ahn & Jin [151], and Shah & Goldstein [152]. The assumptions of normality of the refined ECQ items were also evaluated. None of the observed variables was significantly skewed or highly kurtotic [117,125,126]. No variables had a standardized skewness greater than -1.65, further indicating that all items were normally distributed. Further examination of frequency histograms, expected normal probability plots and detrended normal probability plots also suggested data approximated a normal distribution [117].

**Fig 2:**
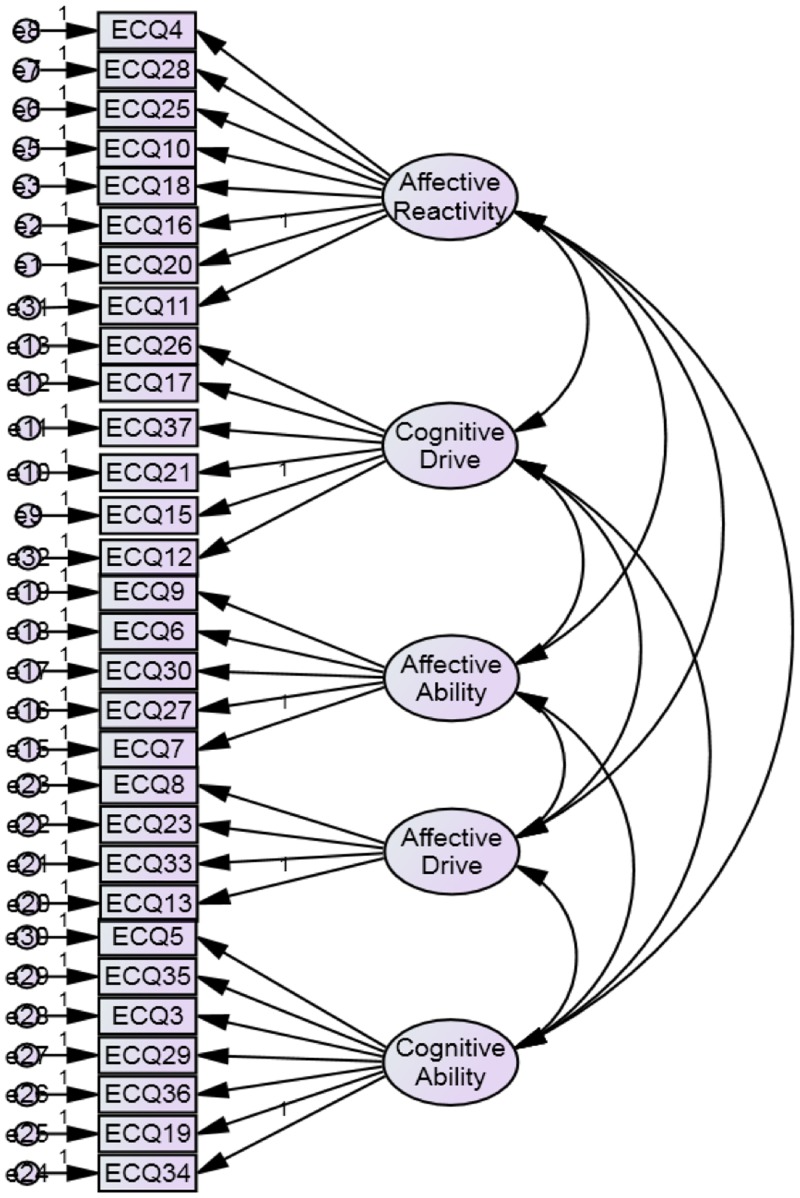
The hypothesized measurement model of the refined ECQ.

Fig 2 represents the initial measurement model tested for the ECQ. The double arrows between cognitive and affective empathy, with further ability and drive components, reflects their covariance.

Numerous tests were used to assess the goodness of fit of the proposed model. Following the recommendations of Tabachnick & Fidell [117], Thompson [122], Cole [153], Cuttance & Ecob [154], Hu & Bentler [155], and Marsh, Balla & McDonald [156], the goodness-of-fit of the ECQ was evaluated using multiple criteria: chi-square χ^2^, the goodness-of-fit index (GFI), the adjusted goodness-of-fit index (AGFI), the comparative fit index (CFI), the root mean square error of approximation (RMSEA), and the standardized root mean square residual (SRMR). For the current study, multiple criteria were used because each index has different strengths and weaknesses in assessing goodness-of-fit between a particular model and the observed data. For instance, the use of chi-square χ^2^ is widely used to assess overall good model fit. However, chi-square χ^2^ is sensitive to sample size, which can lead to inaccurate probability levels and misinterpretations [148,150,155,157]. Due to these issues and deeming it impractical to assess model fit using χ^2^ solely on its own, additional fit statistical tests were developed and included in the current analyses. Based on the recommendations of various researchers within the field [153–156,158,159], the following criteria were used to assess goodness-of-fit of the measurement model of the ECQ: GFI > 0.85, CFI > 0.90 (though > 0.85 is acceptable [160]), AGFI > 0.80, RMSEA < 0.08, and SRMR < 0.08 indicating good model fit [117].

Maximum likelihood estimation was employed to estimate all models. This estimation was appropriate to use given the multivariate normality of the current sample and its appropriate size [117,152]. The adequacy and goodness-of-fit of the overall model was first explored. The chi-square statistic of the first measurement model of the ECQ yielded a statistically significant result, χ^2^ (395) = 754.08, *p* < 0.001. Given the rejection of the null hypothesis and the limitations of chi-square χ^2^, additional and more practical fit indexes were implemented and reviewed in determining the fit of the first measurement model. Some goodness-of-fit tests approached suitable levels (RMSEA = 0.07; PCLOSE = 0.001; SRMR = 0.078), while other goodness-of-fit tests suggested poorer fit (GFI = 0.82; AGFI = 0.78; CFI = 0.82). CFI and AGFI results trended towards acceptable fit.

Post-hoc model modifications were performed in an attempt to develop a better fitting and more parsimonious model. Initially, it was decided to assess the standardized regression weights, also known as factor loadings, in order to amend the measurement model (see Table A in S1 File for standardized regression weights) [150,159]. When first examining the unconstrained estimates in the measurement model of the ECQ, one of the items (ECQ12) was just reaching significance (*p* = 0.05) compared to other significant estimates (*p* < 0.01). The standardized regression weights can be interpreted as the correlation between the observed variable and factor. For this model, the item ECQ12 had a low regression weight estimate of 0.16 loaded onto cognitive drive. It should also be noted the item ECQ11 had a low regression weight estimate of 0.16 loaded onto affective reactivity (*p* = 0.04). This suggests that both items do not highly measure the value dimensions compared to the remaining items loaded onto the identified factors. Instead, both items may be measuring different aspects of social functioning than previously intended. Consequently, both items were removed from the measurement model and the analysis was re-run.

The second measurement model of the ECQ revealed improved model fit (χ^2^ (340) = 611.28, *p* < 0.001; GFI = 0.84; AGFI = 0.80; CFI = 0.86; RMSEA = 0.062; PCLOSE = 0.001; SRMR = 0.075). These results suggest the model fit was tolerable but further improvement to the model could be done. One way to further refine the measurement model was to look at modification indices. AMOS can estimate the improvement in the model fit χ^2^ by freeing a previously fixed parameter to be estimated. Fixed parameters with larger modification indices are the leading candidates in identifying misspecifications of a measurement model in order to improve model fit. By freeing certain fixed parameters through additional paths, these relationships indicate they are estimated rather than fixed. High modification indices of at least 10 should be considered for improving the measurement model fit if there are clear theoretical justifications in doing so [161,162].

The first set of modification indices indicated that a better fit would be obtained if the errors between items ECQ21 (e10) and ECQ17 (e12) were correlated. These items were ‘I am uninterested in putting myself in another’s shoes if I am upset with them’ (ECQ21) and ‘I strive to see how it would feel to be in someone else’s situation before criticizing them’ (ECQ17). Both items theoretically measure the motivation to perspective-take and whether one imputes their own judgments in doing so. Arguably these two items may strongly relate with one another based on the overall tendency in cognitive empathy [11]. This modification index of 12.70 was theoretically justified and consequently applied to the measurement model to be re-run and assessed.

The third measurement model of the ECQ revealed further improved model fit (χ^2^ (339) = 597.23, *p* < 0.001; GFI = 0.84; AGFI = 0.81; CFI = 0.87; RMSEA = 0.06; PCLOSE = 0.02; SRMR = 0.0745). There was also interest in examining the standardized residual covariance matrix. This shows the differences between the sample covariance and the model-implied covariance. With a correct model, most standardized residuals should be less than two in absolute value [150,163]. Byrne [150] argues the better the fit of the model, the smaller the standardized residual covariances. Item EQ29 revealed having the largest standardized residual covariances amongst the measurement model (the largest standardised residual covariance was 3.99 between ECQ29 and ECQ33). This suggests that the model does not adequately estimate the association between these two variables. ECQ29 tended to be problematic for the overall model fit. This item was ‘I am not very good at ‘putting myself in others’ shoes’.’ Although this item was intended to directly assess cognitive ability, there tended to be dissociations between this item and the remaining items on its predicted component. It could be speculated that the abstract wording of ‘putting myself in others’ shoes’ and the negative wording associated with this statement may have confused participants, causing them to respond in a different way than previously intended. Consequently ECQ29 was removed and the model was re-run.

**Fig 3:**
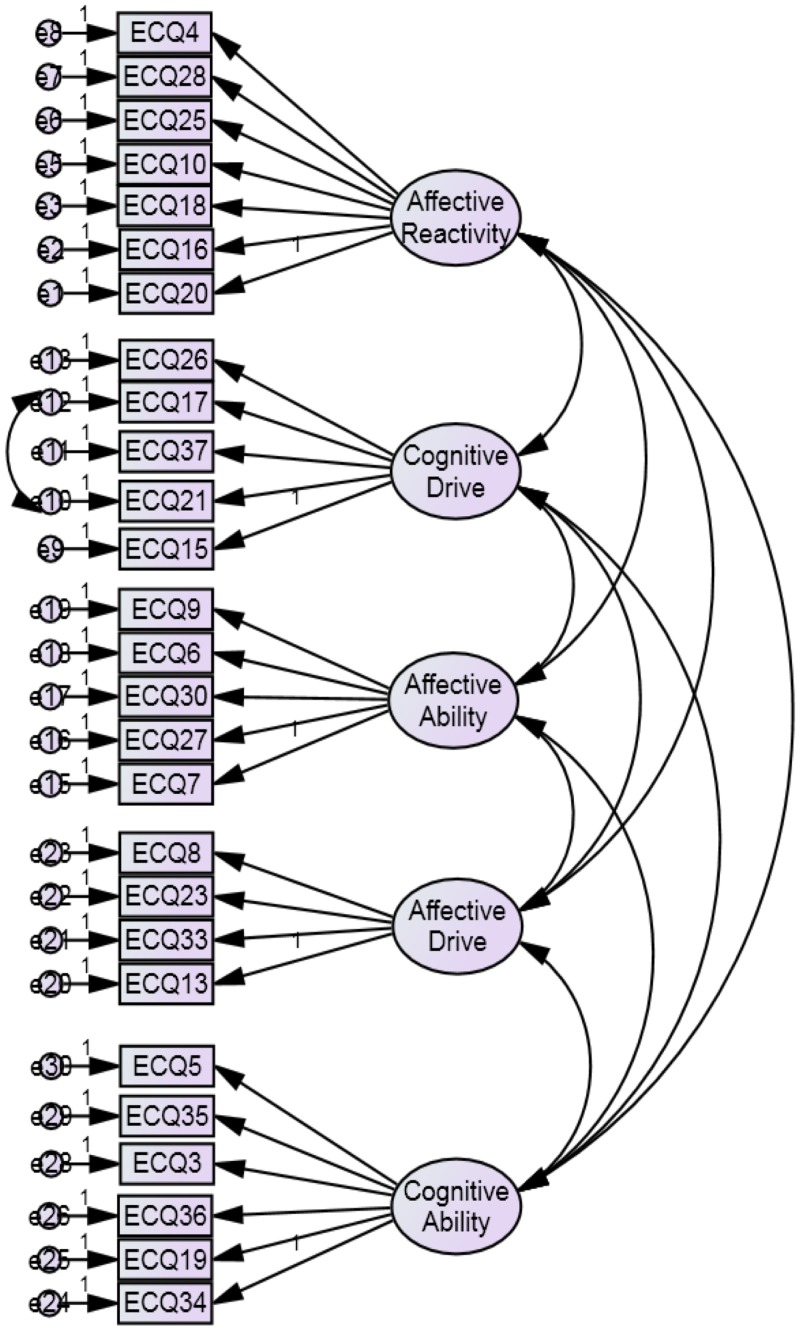
The final measurement model of the refined ECQ.

The fourth measurement model of the ECQ revealed good model fit (χ^2^ (313) = 502.36, *p* < 0.001; GFI = 0.85; AGFI = 0.82; CFI = 0.90; RMSEA = 0.05; PCLOSE = 0.24; SRMR = 0.0642). This model exemplified good fit of the refined ECQ and no further modifications were deemed necessary. See Table 5 for a full outline of goodness-of-fit test results for each measurement model. The final measurement model of the ECQ is presented in Fig 3.

**Table 5:** Goodness-of-fit statistics for all four measurement models of the refined ECQ.

Goodness-of-Fit Tests	Model 1	Model 2	Model 3	Model 4
χ^2^	χ^2^(395) = 754.08**	χ^2^(340) = 611.28**	χ^2^(339) = 597.23**	χ^2^(313) = 502.36**
RMSEA	0.07 (0.06: 0.07)	0.06 (0.05: 0.07)	0.06 (0.05: 0.07)	0.05 (0.05: 0.06)
CFI	0.82	0.86	0.87	0.90
GFI	0.82	0.84	0.84	0.85
AGFI	0.78	0.80	0.81	0.82
SRMR	0.078	0.075	0.0745	0.0642

**p < 0.001

#### Reliability

Correlations, corrected item-total correlations, and Cronbach’s alphas coefficient reliabilities for all items are outlined in Table 6. Cronbach alphas for all subscales were acceptable and demonstrate that they were internally consistent (range = 0.70–0.81). All items also correlated with their respective factor. The correct item-total correlations were also all above 0.30, suggesting all items corresponded with the ECQ overall [114,115]. The Cronbach’s alpha coefficients for the scale if the item were deleted were all within the respected bound, ranging from 0.90 to 0.91 [114,115,164,165]. Findings also showed that none of the items would substantially affect reliability if they were deleted from the overall questionnaire.

**Table 6:** Pearson correlations, corrected item-total correlations and Cronbach’s alpha ∝ coefficient reliabilities of the ECQ.

ECQ Items	2	3	4	5	6	7	8	9	10	11	12	13	14	15	16	17	18	19	20	21	22	23	24	25	26	27
**AR**(1) ECQ4	.35**	.39**	.45**	.53**	0.13	.32**	.37**	.26**	.15*	.28**	.33**	.45**	.26**	.31**	.37**	.43**	.37**	.47**	.25**	.37**	0.13	.24**	0.02	.19**	.24**	.23**
(2) ECQ28	.	.27**	.38**	.25**	.25**	.22**	.23**	.25**	0.02	.28**	0.09	0.09	0.11	0.12	.16*	0.13	.22**	.27**	0.02	.20**	-0.014	-0.027	-0.033	-0.01	0.13	0.04
(3) ECQ25		.	.36**	.28**	.25**	.24**	.44**	.33**	.19**	.27**	.26**	.37**	.22**	.33**	.27**	.26**	.31**	.49**	.22**	.33**	0.13	.18**	0.08	.14*	0.12	0.07
(4) ECQ10			.	.46**	.23**	.22**	.28**	.20**	.15*	.28**	.27**	.29**	.15*	.31**	.38**	.23**	.33**	.46**	.18**	.30**	.18*	.17*	0.1	0.04	.22**	0.12
(5) ECQ18				.	.18**	.28**	.34**	.14*	0.1	.24**	.27**	.42**	.32**	.32**	.45**	.44**	.30**	.32**	.25**	.32**	.23**	.26**	.15*	.23**	.33**	.23**
(6) ECQ16					.	0.1	.27**	.25**	0.11	.16*	0.03	.14*	0.08	0.05	.17*	0.09	.21**	.23**	0.08	.33**	0.02	0.05	0.02	0.05	0.04	-0.02
(7) ECQ20						.	.47**	.22**	.20**	.29**	.25**	.32**	.38**	.26**	.20**	.32**	.29**	.34**	.20**	.28**	.18**	.29**	.24**	.24**	.44**	.26**
**CD** (8) ECQ26							.	.41**	.30**	.34**	.33**	.35**	.35**	.38**	.38**	.44**	.28**	.51**	.43**	.39**	.26**	.16*	.16*	.16*	.35**	.23**
(9) ECQ17								.	.29**	.48**	.36**	.19**	.17*	.20**	.25**	.21**	.31**	.38**	.27**	.33**	0.01	.18**	0.09	0.13	0.11	0.13
(10)ECQ37									.	0.13	.17*	.15*	0.06	0.11	0.13	0.12	.30**	.23**	.22**	.19**	0.08	0.08	0.11	.14*	0.12	0.13
(11)ECQ21										.	.18**	.19**	.20**	.25**	.28**	.15*	.25**	.30**	.31**	.38**	0.11	0.09	.20**	0.09	.25**	.18**
(12)ECQ15											.	.24**	.22**	.22**	.30**	.28**	.27**	.32**	.27**	.22**	0.08	.20**	0.1	.15*	.22**	.21**
**AA** (13) ECQ9												.	.44**	.47**	.41**	.50**	.37**	.39**	.24**	.33**	.38**	.42**	.34**	.31**	.41**	.42**
(14) ECQ6													.	.33**	.39**	.48**	.16*	.26**	.25**	.25**	.35**	.36**	.24**	.17*	.33**	.31**
(15)ECQ30														.	.42**	.48**	.21**	.33**	.22**	.27**	.44**	.42**	.46**	.40**	.58**	.42**
(16)ECQ27															.	.45**	.21**	.32**	.28**	.25**	.23**	.31**	.22**	.19**	.36**	.28**
(17) ECQ7																.	.25**	.35**	.20**	.39**	.38**	.36**	.21**	.28**	.40**	.33**
**AD** (18) ECQ8																	.	.41**	.27**	.37**	0.07	.22**	0.05	.18**	.19**	.19**
(19)ECQ23																		.	.39**	.44**	.20**	.27**	0.13	.26**	.31**	.19**
(20)ECQ33																			.	.27**	.17*	.15*	.22**	.27**	.25**	.32**
(21)ECQ13																				.	0.11	.14*	0.15	0.13	.24**	0.1
**CA** (22) ECQ5																					.	.38**	.47**	.30**	.42**	.43**
(23)ECQ35																						.	.45**	.45**	.44**	.42**
(24) ECQ3																							.	.36**	.53**	.46**
(25)ECQ36																								.	.45**	.35**
(26)ECQ19																									.	.53**
(27)ECQ34																										.
Corrected Item-Total *r*	0.33	0.52	0.52	0.58	0.29	0.52	0.64	0.46	0.31	0.47	0.44	0.65	0.53	0.63	0.59	0.62	0.49	0.64	0.47	0.53	0.44	0.51	0.42	0.43	0.60	0.50
∝ if item deleted	0.91	0.91	0.91	0.90	0.90	0.91	0.91	0.91	0.91	0.91	0.91	0.90	0.91	0.90	0.90	0.90	0.91	0.91	0.91	0.91	0.91	0.91	0.91	0.91	0.91	0.91

**p < 0.01,

*p < 0.05,

AR = Affective Reactivity, CD = Cognitive Drive, AA = Affective Ability, AD = Affective Drive, CA = Cognitive Ability

#### Assessment of the relationship between components of the ECQ

Relationships were examined between all components within the refined ECQ to better understand these components of empathetic behavior (see Table 7). With a Bonferroni adjusted *p*-value of 0.005 (0.05/10), Pearson correlations revealed cognitive ability was positively correlated with cognitive drive (*r* = 0.31, *p <* 0.0001), affective ability (*r* = 0.63, *p* < 0.0001), affective drive (*r* = 0.36, *p* < 0.0001) and affective reactivity (*r* = 0.30, *p* < 0.0001). Cognitive drive was also positively correlated with affective ability (*r* = 0.31, *p <* 0.0001), affective drive (*r* = 0.65, *p <* 0.0001) and affective reactivity (*r* = 0.56, *p <* 0.0001). Similarly, affective ability was positively associated with affective drive (*r* = 0.52, *p* < 0.0001) and affective reactivity (*r* = 0.56, *p* < 0.0001). Lastly affective drive correlated positively with affective reactivity (*r* = 0.61, *p* < 0.0001).

**Table 7:** Pearson correlations between components from the refined ECQ.

Measure	Cognitive Ability	Cognitive Drive	Affective Ability	Affective Drive	Affective Reactivity
Cognitive Ability	-	0.31**	0.63**	0.36**	0.30**
Cognitive Drive		-	0.31**	0.65**	0.56**
Affective Ability			-	0.52**	0.56**
Affective Drive				-	0.61**
Affective Reactivity					-

** *p <* 0.001

#### Sex differences

**Fig 4:**
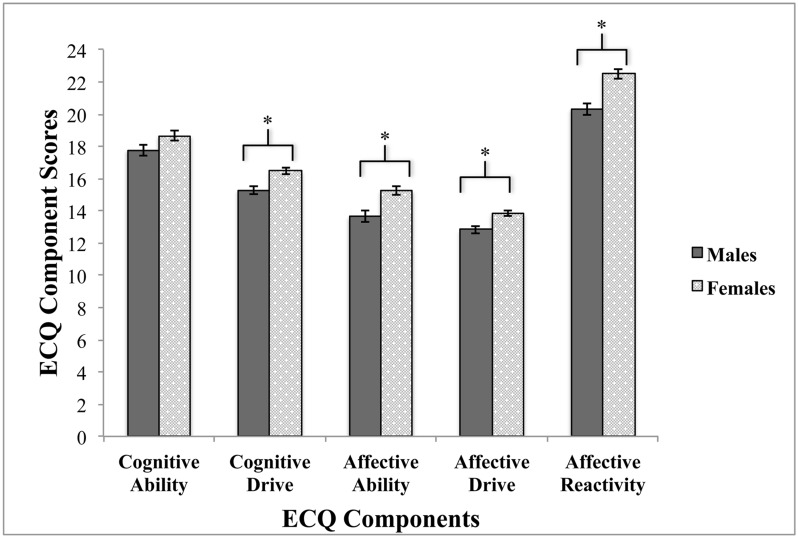
Assessment of sex differences across the five components from the ECQ in 211 participants.

Sex differences in the ECQ were examined on total ECQ scores through an independent t-test. There was a significant difference in total empathy scores between males (mean = 79.88, SD = 12.20) and females (mean = 86.72, SD = 9.65), *t* (209) = -4.55, *p* < 0.0001. Further assessment of sex differences in the ECQ were examined using a between-subjects MANCOVA with sex (male versus female) as the independent variables, age as the covariate, and the five ECQ components as the dependent variables (Table 8). Age was included as a covariate because significant differences between males and females were found for age. The results of the MANCOVA revealed a significant interaction between sex and the five ECQ components with age as a covariate at a multivariate level, Hotelling’s *T* (0.11), *F* (5, 204) = 4.49, *p* < 0.001, partial eta squared = 0.10. Post-hoc univariate analyses revealed a statistically significant effect between sex and scores on cognitive drive (*F* (1, 208) = 10.40, *p <* 0.001, partial eta squared = 0.05), affective ability (*F* (1, 208) = 12.74, *p <* 0.0001, partial eta squared = 0.06), affective drive (*F* (1, 208) = 12.21, *p <* 0.001, partial eta squared = 0.06) and affective reactivity (*F* (1, 208) = 19.66, *p <* 0.0001, partial eta squared = 0.09) when controlling for age. There was also a trending difference between groups on the cognitive ability component when controlling for age (*F* (1, 208) = 2.83, *p* = 0.09) (see Fig 4).

**Table 8:** Total ECQ and component mean scores for 211 males and females.

Measure	Males Mean (SD)	Females Mean (SD)
**Total ECQ**	79.88 (12.20)	86.72 (9.65)
**Cognitive**	33.08 (5.00)	35.13 (4.26)
Cognitive Ability	17.78 (3.42)	18.66 (3.29)
Cognitive Drive	15.31 (2.61)	16.47 (2.12)
**Affective**	46.80 (8.16)	51.59 (6.29)
Affective Ability	13.64 (3.40)	15.25 (2.99)
Affective Drive	12.83 (2.30)	13.84 (1.73)
Affective Reactivity	20.33 (3.73)	22.50 (3.19)

#### Convergent validity

The ranges, means, medians and standard deviations for the SII-SF for both males and females are reported in Table 9. It is worth noting that an inverse square root transformation was applied to the SII-SF scores (see below). Original ranges, means, medians and standard deviations of the untransformed SII-SF scores are described for illustrative and interpretative purposes.

**Table 9:** Ranges, means, medians and SD’s for the SII-SF scores for 95 males and 116 females.

Measure	Range	Mean	Median	SD
SII-SF	23–70	56.44	58.00	8.73
SII-SF—Males	23–70	54.80	57.00	9.74
SII-SF—Females	32–70	57.78	59.00	7.59

Normality for the SII-SF was first assessed through the Shapiro-Wilk test of normality and the examination of histograms (see Figs A and B in S1 File). Findings showed scores for the SII-SF was statistically significant (Shapiro-Wilk = 0.93, *p* < 0.0001). Further examination of a histogram for the SII-SF suggested that scores for the SII-SF exhibited significant negative skew data. SII-SF scores were then transformed using an inverse square root transformation to see whether scores were improved. A follow-up Shapiro-Wilk test of normality revealed the transformed SII-SF was no longer statistically significant (*p* = 0.13). The transformed SII-SF scores were used for the remainder of the analysis. It is worth noting that the interpretation of transformed inverse variables is reversed, where negative relationships are interpreted as positive [114,115].

A two-stage hierarchical multiple regression was conducted to assess construct validity of the five components of the ECQ. The first two-stage hierarchical multiple regression included scores for the SII-SF as the dependent variable. Sex and age were entered at stage one of the regression model, and the five empathy components were entered at stage two (see Table 10).

**Table 10:** A two-stage hierarchical multiple regression of demographics and ECQ components predicting SII-SF scores.

SII-SF Scores+					
Step	Predictor	β	*t*	*F*	Sig.
**1**				3.38 (2, 208)	0.04*
	Sex	-0.14	-2.07*		
	Age	-0.08	-1.17		
**2**				14.93 (7, 203)	0.00**
	Sex	0.01	0.21		
	Age	-0.05	-0.89		
	Cognitive Ability	-0.18	-2.46*		
	Cognitive Drive	-0.04	-0.49		
	Affective Ability	-0.11	-1.31		
	Affective Drive	-0.32	-3.80**		
	Affective Reactivity	-0.08	-0.95		

*p ≤ 0.05,

** *p* ≤ 0.01,

^+^please note inverse score transformation interpretation

Findings revealed that at stage one, sex contributed significantly to the regression model, *F* (2, 208) = 3.38, *p* < 0.05, with an adjusted *R*^2^ of 0.02. Introducing the five empathy components from the ECQ explained an additional 30% of variation in SII-SF scores, and this change in the adjusted *R*^2^ was statistically significant, *F* (7, 203) = 14.93, *p <* 0.0001, with cognitive ability (β = -0.18, *p* < 0.01) and affective drive (β = -0.32, *p* < 0.0001) as significant predictors SII-SF scores.

### Discussion

The results using CFA confirmed the five-factor model of the ECQ consisting of cognitive ability, cognitive drive, affective ability, affective drive and affective reactivity, which demonstrated that the five-factor solution provides a good fit, especially after improving the wording of items within the ECQ. Results also showed that females scored higher than males on four out the five components of the ECQ. Females self-reported higher scores on all three affective components of empathy measured through the ECQ compared to males. In addition, there were significant differences on the cognitive drive component and a trending difference on the cognitive ability component. These findings are in-line with extensive evidence that females score significantly higher than males on self-report measures of empathy [7,16,24,96,105,106]. It is worth noting that there were larger sex differences on the affective components of empathy. This is in-line with some literature suggesting minimal sex differences between males and females on measures of cognitive empathy, particularly in cognitive empathic ability [6,11,106]. Findings also revealed higher scores in affective drive predicted higher scores on the SII-SF. This showed that the interest or tendency to be sensitive to others’ emotions relates to a greater sense of feeling towards others [166–168]. Findings also showed higher scores in cognitive ability predicted higher scores on the SII-SF. This suggested that the ability or skill to take another’s perspective relates to having a greater interest in others socially, showing evidence of convergent validity of the ECQ.

## General Discussion

The aim of the present research was to develop and validate the ECQ, a new brief self-report measure of empathy that produces a total empathy score, as well as scores for a number of relevant components within empathy. The results of study one using a PCA revealed a five-factor model of the ECQ consisting of the components of cognitive ability, cognitive drive, affective ability, affective drive and affective reactivity, and this model was then confirmed using a CFA on an independent sample. The results also showed significant sex differences for all components of affective empathy, with minimal difference between sexes evident in cognitive empathy. Convergent validity was also provided for the ECQ, as scores on the affective drive component of empathy predicted scores on the SII-SF measure of social interest, but not affective ability scores. Cognitive ability scores also predicted scores on the SII-SF. Together, the present results validate the ECQ as a new measure of empathy that indexes various relevant components to provide a useful research tool for investigating empathy in a healthy population and in future in various clinical populations.

Empathy theory and research has proposed a multidimensional construct of empathy comprising of cognitive and affective aspects, as well as drive and ability within these components [8,9,11,12,14,17,57,58,60,64,169]. The ECQ provides a 27-item self-report tool that is brief and easy to implement for research investigating empathy and its components, including the ability versus the drive to empathize. These further components are not currently indexed by any questionnaire measure of empathy to date. Cognitive ability and cognitive drive were distinguished in the ECQ by using certain wording and phrases in Study one. Interestingly, the factor corresponding with cognitive ability included items derived primarily from the EQ and EQ-short, whereas the cognitive drive factor involved mainly items derived from the IRI. This supports the idea that the EQ and EQ-short may measure more about people’s awareness about their abilities in empathizing, whereas the IRI may capture more about peoples drive towards empathizing with others. The ECQ goes further by indexing both drive and ability aspects of empathy, including cognitive empathy, together within the same questionnaire.

Findings also revealed a further fifth component of empathy interpreted as affective reactivity. Affective reactivity is argued to be action-specific by individuals responding to another’s emotional experiences which often entails sharing these emotions and feelings [7,8,12–14,23,24,56]. These reactions may be elicited by the initial recognition or sensitivity to other’s feelings and emotions [16,32]. One may have an ability to recognize and be sensitive one’s emotions, a key component of affective empathy [9,11,83], however these swift abilities and drives may then translate to react or respond to other’s emotional experiences [16]. It is suggested that the development of representations of others’ feelings and emotions through the recognition, observation and sensitivity towards emotions, such as emotion recognition and contagion, allows the perceiver to produce the same feelings and ultimately allows the observer to respond appropriately [7–9,16,32,42,52,170]. For instance, evidence suggests that increased sensitivity towards feelings and emotions of others tends to elicit overwhelmed emotional responses [171]. Additional studies have shown that the recognition of emotions, such as fear and happy faces, tend to produce activation of the IFG, suggesting observers are engaged and share the same feeling as the target [172,173]. This suggests that the initial recognition and sensitivity towards emotions and feelings act as precursors and ignite emotional responses [9,14,30,32]. The current findings showed that items loaded onto the affective reactivity factor were separate from items loaded onto affective ability or affective drive, suggesting that there is a further distinction within the affective empathy component. Hence, wording may capture the initial recognition and sensitivity of emotions and feelings but not necessarily ignite emotional sharing.

The present research also showed that females self-reported having greater empathy overall compared to males, which is consistent with extensive research reporting that females score higher than males on various questionnaire measures of empathy [7,16,24,96,105,106]. The female advantage in self-reporting higher empathy is also consistent with empirical research involving behavioral tasks of empathy, which have generally reported that females outperform males. For example, females are better able than males in reading the mental and emotional states of others from only their eye regions [65,66,174], and in recognizing faux pas situations [175]. Neuroimaging studies have also shown sex differences in brain activations during empathy-related tasks. For example, greater mirror neuron activity is seen in females versus males while evaluating the emotional states of the faces of others [176] and there are enhanced longer-latency ERP responses in females compared to males to viewing other people in pain [177]. Therefore, the female advantage in self-reporting about empathy is consistent with greater performance by females in both self-report, behavioral tasks and neuroimaging studies of empathy.

Interestingly, the female advantage in empathy in the present research with the ECQ was most apparent across the affective components, including affective reactivity. However, there were minimal sex differences evident in the cognitive components of the ECQ. These results show that females more naturally tune into the emotional states of others and are more likely to react and respond to other’s emotions and feelings [7,8,12–14,16,19,23,24]. Similar findings showing a significant sex difference on aspects of affective empathy through self-report measures were found in Muncer & Ling [61], Michalska, Kinzler & Decety [96], and Rueckert, Branch & Doan [106]. For instance, Muncer & Ling [61] argue sex differences in self-reported affective empathy may be related to increased drive rather than ability, which may relate to higher levels of neurosis in females as seen in relationships between measures of emotional intelligence and neurosis [55,178,179]. The idea that females’ beliefs about their own motivations and tendencies to emotionally react and respond to other’s feelings may drive females to respond more empathically is also in-line with the works of Ickes, Gesn & Graham [55] and Klein & Hodges [102]. Klein and Hodges [102] examined perceived and actual empathic accuracy between males and females and found that when controlling for motivation, these sex differences were no longer present.

However the minimal sex differences on the cognitive components of empathy suggest the sexes are less different to each other in the drive and ability to adopt the perspectives of others. This is in-line with other literature using questionnaires of empathy reporting little or no sex differences on some measures of cognitive empathy (e.g. [6,11,61,106]). From these results it appears that females and males mostly differentiate from each other when considering the emotions of others, which also provides additional support these components of empathy are at least partially dissociable from one another. For instance, when developing the IRI, Davis [11,83] found smaller sex difference among the four components of the IRI was among the perspective-taking subscale. Additional researchers, such as Rueckert, Branch & Doan [106], examined empathy amongst males and females and found only sex differences on components assessing affective empathy and not cognitive empathy. This result shows sex differences in empathy may be greater in affective empathy using self-report measures, which further supports the possibility that these differences could be due to females’ beliefs about their own abilities and drives to share and react to others’ emotions.

Convergent validity of the ECQ was also established in Study two, with higher scores on the affective drive component of the ECQ associated with scores on the independent measure of social interest (SII-SF). This provides some evidence that the drive component of the ECQ is effectively indexing the interest to empathize with others, as both affective drive and SII-SF include items about socially engaging with others. Scores on the cognitive ability component were also associated with scores on the SII-SF, although cognitive drive did not show any association to the SII-SF. No other components from the ECQ predicted scores on the SII-SF. This finding is in-line with evidence suggesting that empathy is partially dissociable, and cognitive and affective empathy may be needed for the same process, while utilizing different regions of the brain for each component [52]. These further results may have been due to the nature of the wording of items within the SII-SF, because some of the items in this scale may reflect more general cognitive abilities. An example item is “My plans generally turn out the way I want them too”. Another potential explanation could be that some aspects of both affective and cognitive components may be involved in the drive to engage socially with others, such that greater ability in predicting and understanding the behavior of others may lead to higher interest in socializing. Future work may consider validating the ECQ with additional behavioral measures of empathy to further understand this discrepancy.

### Limitations

There are some limitations of these studies to be noted. The sample in Study one comprised of students and staff at the University of Bath, which may potentially limit the generalizability of the results to the wider population. However, as previously mentioned, the sociodemographic nature of the sample outlined in Study one was similar to that of other sociodemographic profiles in comparable empathy studies within the literature [16,107]. Study two also included additional online recruitment techniques to access a larger and more diverse sample beyond the scope of the University of Bath sample. Future works should include further replicating and cross-validating the ECQ in an independent and diverse sample. The ECQ is also a self-report measure of empathy, and the scores might be affected by factors such as social desirability and response biases, so participants’ actual empathic behavior may be somewhat different to their ECQ scores. However, some evidence shows that participants respond in similar ways, particularly with sex differences. Some findings suggest that females tend to score higher than males on behavioral measures of empathy, such as false belief tasks and emotion recognition ([65,66,174,175,180–182]; for a review, see [104]). In order to further validate the ECQ, future work should compare participant’s self-perceptions of their own empathy indexed by the ECQ with additional independent behavioral measures of empathy.

### Conclusion

The ECQ is a new quick and easy to implement measure of empathy that assesses further components of empathy than previous questionnaires, including cognitive ability, cognitive drive, affective ability, affective drive, and affective reactivity. This five- component model was identified using PCA and then confirmed using CFA in an independent sample. Results further showed that females scored higher than males on four out of the five empathy components, and that the affective drive and cognitive ability components predicted scores on an independent measure of social interest. Together, the present results demonstrate the ECQ to be a valid measure of wider components of empathy, which is consistent with more recent models and ideas about delineating empathy. The ECQ provides a valuable tool for assessing empathy in the general population.

## Supporting Information

S1 File Additional analyses and copy of ECQ questionnaire. (DOCX)

S1 Data Study one dataset. (XLSX)

S2 Data Study two dataset. (XLSX)
