# Supplementary material for: The Development and Validation of the Empathy Components Questionnaire (ECQ)
Source: PLoS One. 2017 Jan 11;12(1):e0169185. doi: 10.1371/journal.pone.0169185 (PMC5226785; doi:10.1371/journal.pone.0169185)
Supplement: S1 File — (DOCX) [file pone.0169185.s001.docx]

Supporting Information

Table A. Standardized regression weights in the initial measurement model of the refined ECQ

| **Item** |  | **Factor** | **Estimate** |
| --- | --- | --- | --- |
| ECQ20 | <--- | Affective_Reactivity | .472 |
| ECQ16 | <--- | Affective_Reactivity | .319 |
| ECQ18 | <--- | Affective_Reactivity | .634 |
| ECQ10 | <--- | Affective_Reactivity | .631 |
| ECQ25 | <--- | Affective_Reactivity | .592 |
| ECQ28 | <--- | Affective_Reactivity | .445 |
| ECQ4 | <--- | Affective_Reactivity | .719 |
| ECQ15 | <--- | Cognitive_Drive | .492 |
| ECQ21 | <--- | Cognitive_Drive | .529 |
| ECQ37 | <--- | Cognitive_Drive | .389 |
| ECQ17 | <--- | Cognitive_Drive | .600 |
| ECQ26 | <--- | Cognitive_Drive | .726 |
| ECQ7 | <--- | Affective_Ability | .686 |
| ECQ27 | <--- | Affective_Ability | .609 |
| ECQ30 | <--- | Affective_Ability | .710 |
| ECQ6 | <--- | Affective_Ability | .572 |
| ECQ9 | <--- | Affective_Ability | .713 |
| ECQ13 | <--- | Affective_Drive | .602 |
| ECQ33 | <--- | Affective_Drive | .504 |
| ECQ23 | <--- | Affective_Drive | .744 |
| ECQ8 | <--- | Affective_Drive | .555 |
| ECQ34 | <--- | Cognitive_Ability | .670 |
| ECQ19 | <--- | Cognitive_Ability | .750 |
| ECQ36 | <--- | Cognitive_Ability | .557 |
| ECQ29 | <--- | Cognitive_Ability | .442 |
| ECQ3 | <--- | Cognitive_Ability | .651 |
| ECQ35 | <--- | Cognitive_Ability | .645 |
| ECQ5 | <--- | Cognitive_Ability | .620 |
| ECQ11 | <--- | Affective_Reactivity | .162 |
| ECQ12 | <--- | Cognitive_Drive | .155 |

A) B)


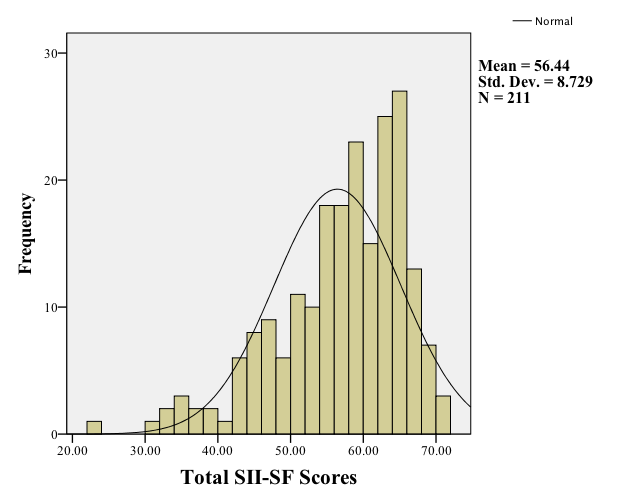

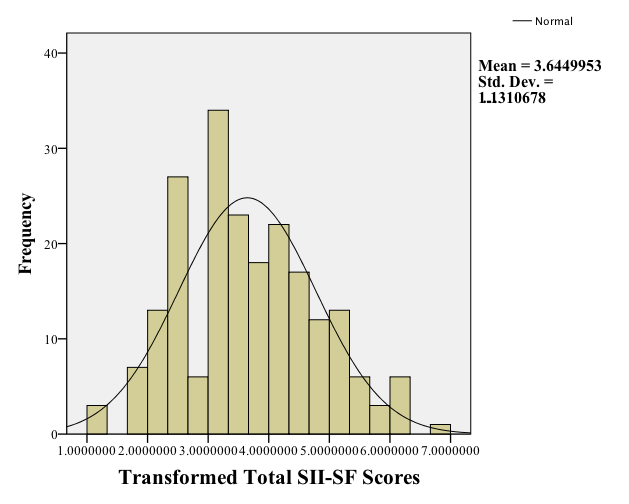


Figs A) Normality assessment of total SII-SF scores through a histogram in 211 participants; B) Normality assessment of inverse square root transformation total SII-SF scores through a histogram in 211 participants

Text A. The 27-item Empathy Components Questionnaire (ECQ)

1. I am usually successful in judging if someone says one thing but means another. **(CA)**

2. When someone seems upset, I am usually uninterested and unaffected by their emotions. **(AR)**

3. I am not very good at predicting what other people will do. **(CA)**

4. My friends often tell me intimate things about themselves as I am very helpful. **(AA)**

5. I am good at responding to other people’s feelings. **(AA)**

6. I am not interested in protecting others, even if I know they are being lied to. **(AD)**

7. I am not very good at helping others deal with their feelings. **(AA)**

8. Others’ emotions do not motivate my mood. **(AR)**

9. I have a desire to help other people. **(AD)**

10. When talking with others, I am not very interested in what they might be thinking. **(CD)**

11. I feel pity for people I see being bullied. **(AR)**

12. I strive to see how it would feel to be in someone else’s situation before criticizing them. **(CD)**

13. I avoid getting emotionally involved with a friend’s problems. **(AR)**

14. I do well at noticing when one of my friends is uncomfortable. **(CA)**

15. I like to know what happens to others. **(AR)**

16. I am uninterested in putting myself in another’s shoes if I am upset with them. **(CD)**

17. When I do things, I like to take others’ feelings into account. **(AD)**

18. I am not always interested in sharing others’ happiness. **(AR)**

19. I like trying to understand what might be going through my friends’ minds. **(CD)**

20. I am poor at sharing emotions with others. **(AA)**

21. When someone is crying, I tend to become very upset myself. **(AR)**

22. I don’t intuitively tune into how others feel. **(AA)**

23. I avoid thinking how my friends will respond before I do something. **(AD)**

24. I am not very good at noticing if someone is hiding their emotions. **(CA)**

25. During a conversation, I’m not very good at figuring out what others might want to talk about. **(CA)**

26. I am good at sensing whether or not I am interrupting a conversation. **(CA)**

27. I take an interest in looking at both sides to every argument. **(CD)**

**ECQ Scoring Key**

***The scoring ranges from 1 (strongly disagree) to 4 (strongly agree) for the following items:*** 1, 4, 5, 9, 11, 12, 14, 15, 17, 19, 21, 26, 27.

***The scoring range is reversed from 1 (strongly agree) to 4 (strongly disagree) for the following items:*** 2, 3, 6, 7, 8, 10, 13, 16, 18, 20, 22, 23, 24, 25.

**The following items are used to compute the score for each component (reverse-scored items are in bold):**

**Cognitive ability (6 items):** 1, **3**, 14, **24**, **25**, 26

**Cognitive drive (5 items): 10**, 12, **16**, 19, 27

**Affective ability (5 items):** 4, 5, **7**, **20**, **22**

**Affective drive (4 items): 6**, 9, 17, **23**

**Affective reactivity (7 items): 2**, **8**, 11, **13**, 15, **18**, 21

Summing the component scores for cognitive ability and cognitive drive produces a cognitive empathy score. Summing the component scores for affective reactivity, affective ability and affective drive produces an affective empathy score. Summing the component scores for cognitive ability and affective ability provides a total empathic ability score. Summing the component scores for cognitive drive and affective drive provides an empathic drive score. The sum of cognitive and affective component scores produces the cumulative total empathy score.
